# Hospital Meetings

**Published:** 1905-05-20

**Authors:** 


### HOSPITAL MEETINGS.

### BRITISH HOME AND HOSPITAL FOR INCURABLES, STREATHAM.

The forty-fourth annual meeting was held at the British Home and Hospital for Incurables on the 11th inst., at 3 o'clock, Earl Amherst, who is president, being in the chair.

The names of successful candidates as inmates and receivers of pensions having been read out, the president directed the reading of the annual report and followed it by a short speech on the financial position of the hospital. There is a debt to the bank of £2,000, and an effort is being made, by means of a shilling collection, to clear off this debt. Every month the sum of £600 is paid in pensions alone, and of late the pressure on the hospital's funds has been such that legacies, instead of being funded, have been used to make up the necessary annual income. The Board feel that this is very unsatisfactory and trust that a generous public will come forward to help them. There is a great need for more subscribers. Generous friends are removed by death and their place is not always filled by others.

Two members of the Board, Brigadier-General Eyre-Crabbe and Mr. Walter Moresby Chinnery have died during the past year, and their kind services are much regretted by their colleagues.

Proceedings terminated with a vote of thanks to Lord Amherst for presiding.

